# Anterior accessory great saphenous vein as a cause of postoperative recurrence of veins after radiofrequency ablation

**DOI:** 10.25122/jml-2021-0318

**Published:** 2022-04

**Authors:** Yrij Svidersky, Volodymyr Goshchynsky, Bogdan Migenko, Liudmyla Migenko, Oleg Pyatnychka

**Affiliations:** 1. Department of Surgery, Institute of Postgraduate Education, I. Horbachevsky Ternopil National Medical University, Ternopil, Ukraine; 2. Second Department of Internal Medicine, I. Horbachevsky Ternopil National Medical University, Ternopil, Ukraine

**Keywords:** RFA, anterior accessory great saphenous vein, postoperative recurrence of veins, RFA – radiofrequency ablation, CEAP – Clinical-Etiological-Anatomical-Pathophysiological, AAGSV – anterior accessory great saphenous vein, SFJ – saphenofemoral junction, SSV – small saphenous vein, SPJ – saphenopopliteal junction, QoL – quality of life

## Abstract

There are problems of postoperative relapse of veins after radiofrequency ablation (RFA). The study aims to analyze the causes of postoperative recurrence of veins after RFA. 928 patients with varicose veins of the lower extremities, clinical classes C_2_-C_4_ according to the CEAP classification, were treated in the ambulatory surgery centers using RFA. The causes of varicose recurrence showed that it was caused by: a) high fusion of the anterior accessory great saphenous vein (AAGSV) with great saphenous vein (GSV) directly in the saphenofemoral junction (SFJ), which was not revealed by preoperative ultrasound (1.7%); a long stump of the GSV after the RFA (7.8%); progression of varicose disease with the small saphenous vein (SSV) and formation of new reflux associated with insufficiency of the saphenopopliteal junction (SPJ) (4.7%); d) insufficiency of perforating veins of the tibioperoneal group (Sherman, Boyd), as well as Gunter (3.8%); e) neovascularization with dilation of small vessels in the area of the saphenofemoral junction (0.97%). A comparative assessment of the quality of life (QL) after different surgery methods 3 years after implementation was carried out. Thus, QL in all patients who underwent surgery significantly improved than before surgery. However, after the RFA GSV+AAGSV, the patients had better QL by all scales than those who underwent only RFA GSV. Operations performed simultaneously on GSV and AAGSV have better functionality than GSV-only RFA.

## Introduction

Low trauma and cosmetic effect of radiofrequency ablation (RFA) is now a priority in treating varicose vein disease of the lower extremities in outpatient care [1–5]. The specificity of radiofrequency ablation of the great saphenous vein (GSV) does not involve crossectomy. The recommended location of the ClosureFAST catheter head is up to 2 cm distal to the terminal valve. Therefore, during this surgery, its stump of different lengths may remain [6]. It is established that the venous system is a network of interconnected vessels; any preserved inflow of the saphenous-femoral mouth can cause postoperative recurrence. However, according to the literature, the greatest risk of recurrence is associated with the stump of GSV of venous tributaries, including great venous trunks parallel to the GSV in the thigh; these are the anterior tributaries [7–10]. In clinical practice, the anterior accessory great saphenous vein is of the greatest interest. The AAGSV is defined as any vein that accompanies the great saphenous vein and is located on the anterior surface of the thigh superficially to the great saphenous vein and is not surrounded by a saphenous fascial sheath. In 41%, the AAGSV flows into the great saphenous vein 1 cm distal to the saphenofemoral mouth [11].

It should be noted that due to the introduction of new treatments for the varicose disease of the lower limbs, in particular endovasal thermal vein ablation, the REVAS recommendations for postoperative recurrence of VD have no relevance any more [8, 9]. Therefore, to change these recommendations, a new concept of the PREVAIT – PREsence of Varices (residual or recurrent) After InTervention was adopted [12–14]. According to the recent literature, the cause of venous recurrence after RFA may be new reflux in a complete or partial recanalized segment of the great or small subcutaneous vein, new reflux in the anterior accessory great saphenous vein, in the perforating veins [8, 9, 13–15].

Current research emphasizes postoperative reflux of GSV and small saphenous vein (SSV) and lesser reflux from the perforating veins of the thigh and lower leg. At the same time, the significance of the anterior accessory great saphenous vein (AAGSV) in the development of recurrence has not been sufficiently studied, especially in the absence of reflux [8, 9, 14–16]. It should be emphasized that multicenter research points out possible recurrence in AAGSV in the late postoperative period (in 1–3 years). Unfortunately, we have not found in the literature any clear information on the "exclusion" of AAGSV from the bloodstream in the absence of reflux, the optimal location of the ClosureFast catheter for RFA in the proximal part of GSV, as well as the parameters of the COVIDIEN device, produced by Medtronics, in the mouths of more than 13–16 mm. We are convinced that these issues are significant for reducing the number of postoperative recurrences of varicose disease.

## Material and Methods

**Figure 1.**
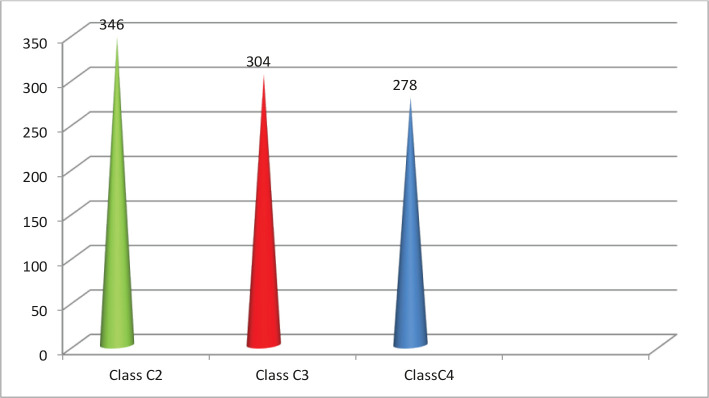
The number of patients by clinical features (the CEAP classification).

From 2016 to 2020, in the ambulatory surgery centers of Zhytomyr, Ternopil, and in the City Phlebological Centre at the Department of Surgery of the Faculty of Postgraduate Education of Ternopil National Medical University, 928 patients with varicose veins of the lower extremities, clinical classes C_2_–C_4_ according to the CEAP classification were treated using RFA. Clinical classes C_2_–C_4_ comprised: class C_2_ – 346 patients, class C_3_ – 304 patients, and class C_4_ – 278 patients (Figure 1). According to the age classification of the World Health Organization, the patients were distributed as follows: 25–44 years (young age) – 389 (41.9%) patients; 44–60 years (average age) – 301 (32.2%) patients; 60–75 (elderly) – 179 (19.3%) patients; more than 75 years (old age) – 59 (6.4%) patients. The mean comorbidity index, according to Charlson, was 3.4±0.6. The comorbidity index was associated with the age of patients (25.7% over 60 years) and the presence of one or two comorbidities (hypertension, coronary heart disease, diabetes, chronic obstructive pulmonary disease).

Exclusion criteria were: a) infectious and inflammatory diseases of the soft tissues of the lower extremities; b) deep vein thrombosis of the lower extremities; d) trophic ulcers of the lower extremities at the time of the study; e) obliterating diseases of the aorta and arteries of the lower extremities.

All patients underwent ultrasound examination to establish phlebo hemodynamic disorders in the limb. Ultrasound of the venous system of the lower extremities was performed using the Mindray Z5 (produced by Mindray Bio – MedicalElectronics, CO, China) with a sensor frequency of 5–14 MHz and the corresponding standard software package by this company for venous investigations. The patients were examined in the afternoon in vertical and horizontal positions. To determine the strategy and tactics of radiofrequency vein ablation using ultrasound, we studied: a) the patency of deep veins and the state of their valvular apparatus; b) the presence of reflux in the saphenofemoral and sapheno-popliteal junction, its duration, c) the presence of reflux in the upper, middle and lower third of the thigh, its duration; e) the diameter of the GSV under the valve, as well as the diameter of the GSV trunk; f) anatomical and topographic features of the sapheno-femoral junction; g) features of the location of the anterior tributaries of the GSV and the presence of reflux in them; g) the state of the perforating veins in the thigh and lower leg. It should be emphasized that duplex ultrasound for patients with varicose disease should always be included in the AAGSV investigations before surgical treatment.

If the presence of venous reflux was determined after sonography of the sapheno-femoral junction area, the patients were divided into three groups: the 1^st^ group – the presence of AAGSV reflux in the competent sapheno-femoral junction (SFJ); the 2^nd^ group – reflux in incompetent SFJ and AAGSV; the 3^rd^ group – reflux only in SFJ and GSV in competent AAGSV. In our opinion, such an ultrasound examination is very important for choosing tactics in VD treatment.

To eliminate the vertical shunt, the RFA was used according to the VNUS – ClosureFAST method, using a COVIDIEN device by Medtronics. In 242 (26.1%) patients, the RFA of the initial segment of GSV was performed at a diameter of 16±0.4 mm.

The effectiveness of the surgeries was evaluated using clinical and ultrasound pictures before the surgery, in 3, 6, 12 months, and in 1, 2, 3 years after it. The clinical severity of venous diseases was determined by the venous clinical severity score (VCSS), venous segmental disease score (VSDS), and physical activity by the venous disability score (VDS). Objective assessment of the severity of chronic venous disease was performed by the venous severity scoring (VSS). To objectively assess the impact of VSS surgeries and quality of life, the patients were divided into 2 groups: the 1^st^ group – 115 patients who underwent only RFA GVS; the 2^nd^ group – 131 patients who underwent simultaneous RFA GVS and AAGSV.

The quality of life (QL) was assessed using the general quality of life questionnaire SF-36 (the Short Form-36). It allowed assessing the dynamics of recovery of patients' physical, psychological, and social functioning after surgery. This test consisted of 36 questions that formed 8 scales: physical functioning (PF), role-physical functioning (RP), bodily pain (BP), and general health (GH): vitality – (VT), social functioning (SF), role emotional (RE), mental health (MH). The patients filled in the questionnaire 3 years after the surgery. The answers to the questions were evaluated by a nominal scale from 0 to 5 points, and then the overall score for each statement was determined.

The obtained results were statistically processed using the package of statistical functions of the Microsoft Excel 2010 computer program on a personal computer by the statistical variation method of analysis. The arithmetic mean (X), the standard error of the arithmetic mean (m), the normal deviation (t), and the correlation coefficient (r) were calculated. The probability level (p) was established by testing hypotheses on the equality of the centres of distribution of two samples (t – Student's t-test), the normal-Laplace (NL) distribution, and the sign test. In other cases, the Mann-Whitney U-test (differences at p <0.05 were statistically significant) was used.

## Results

According to the ultrasound examinations, the diameter of the GSV at the mouth area in case of terminal valve failure directly depends on the clinical stage of varicose disease. Thus, in 391 (42,1%) patients with clinical stage C_2_, the average diameter of the vein was 9.1±2.3 mm, C_3_–C_4_ -12.9±2.8 mm (375 – 40.4%), and C_5_–C_6_ -14.3±3.5 mm in 162 (17.5%) patients, respectively. This is important for choosing thermal energy power in laser or radiofrequency vein ablation to prevent a postoperative recurrence.

Ultrasound diagnosis of the AAGSV location is equally important for preventing VD recurrence. It should be noted that AAGSV is not only a tributary of the sapheno-femoral junction but one of the subcutaneous trunks located in the subcutaneous thigh lateral to the GSV. AAGSV ultrasound was performed for the patients standing. According to our studies, the AAGSV was located laterally from the GSV and medially to the wall artery. It flowed into the GSV up to 1.0±0.5 cm distal to the SFJ. The ultrasound examination of the AAGSV from the groin to the lower third of the thigh showed the GSV more medial and evidenced below the knee, while the AAGSV was short and was detected anteriorly and laterally from the GSV, mid to distal thigh. The length of the AAGSV was 5–25 cm (median – 13.2). According to examination results, the competent AAGSV had a diameter of 3.1±0.1 mm, and 5.2±0.6 mm at reflux in the AAGSV.

**Figure 2.**
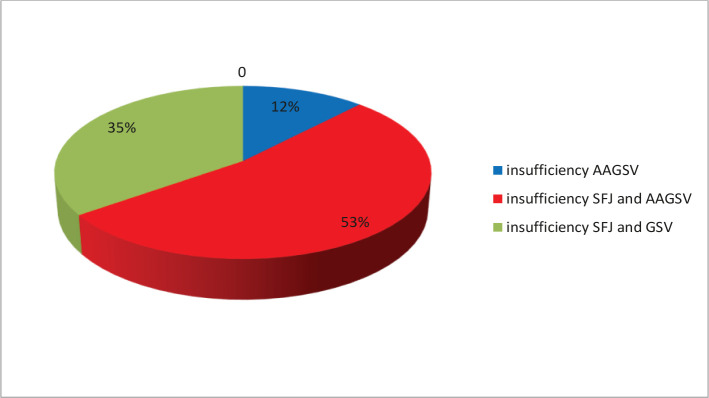
Ultrasound examination results of AAGSV insufficiency, SFJ before radiofrequency ablation of veins.

Ultrasound diagnosis showed that among 928 examined patients, 12% (n=111) had isolated valvular insufficiency of the AAGSV (the 1^st^ group of patients); in 53% of cases (n=492) – combined valvular insufficiency of SFJ and AAGSV was revealed (the 2^nd^ group of patients); in 35% (n=325) patients (the 3^rd^ group) – valvular insufficiency only of SFJ and GSV (Figure 2).

### Features of surgery in cases of AAGSV insufficiency

The indications for radiofrequency ablation of the AAGSV in cases of insufficiency were: the presence of reflux ≥0.5 sec., diameter 5.2±0.6 mm, direct course of the AAGSV, the absence of its expanded inflows in the lower third of the thigh. Cannulation of the subfascial part of the AAGSV was performed under ultrasound control at the lowest point of reflux; usually, it was the middle third of the anterior surface of the thigh. The ablation catheter was passed into the AAGSV to flow into the GSV (with sonographer control). During the radiofrequency ablation (n=38), a tumescent anesthetic was administered. The average length of the coagulated AAGSV was 10.4±5.2 cm. In cases of varicose veins in the projection of the AAGSV, the RFA (middle, lower third of the thigh) was accompanied by the Varadi mini phlebectomy (n=31) or foam sclerotherapy with 1% foam polidocanol (n=28). In cases when it was impossible to pass a catheter into the AAGSV (diameter of up to 3.1±0.1 mm, uneven course of the subfascial part, tortuosity), ultrasound-guided foam sclerotherapy (UGFS) was performed (14 patients).

In combined valvular insufficiency of the SFJ and AAGSV (n=492), two introducers were located at distal points, and reflux was determined in the GSV and AAGSV. Radiofrequency ablation was performed alternately using infusion with a tumescent anesthetic, first for the GSV and then for the AAGSV (according to the technique described). In cases of insufficiency of only the SFJ and GSV (n=325), a standard RFAwas performed, placing the introducer at the distal point of reflux.

During the 1^st^ year of the follow-up, a post-procedure ultrasound examination of the veins revealed recurrence of VD in AAGSV in 5 (4.5%) patients from the 1^st^ group. However, two years later, 23 (20.7%) patients had a visual recurrence of AAGSV VD, mainly in the middle and lower third of the thigh. In our opinion, it was caused by insufficiency of the oscillation valve and valves in the GSV in 17 (15.3%) patients. In 6 (5.4%) cases, the recurrence was caused by the progression of varicose disease in the SSV. Postoperative recurrence was eliminated by RFA GSV and SSV in combination with the Varadi mini phlebectomy and varicose vein puncture sclerotherapy with 0.5% polidocanol solution.

During the first year after surgery in the 2^nd^ group of patients (reflux in the incompetent SFJ and AAGSV), the recurrence of varicose disease due to AAGSV failure was observed in 12 (2.4%) patients. After 2 years, partial recanalization of the GSV in the lower thigh occurred in 20 (4%) of 492 patients. After 2 years, new reflux in the AAGSV was evidenced in 11 (2.2%) cases and after 3 years – in 19 (3.9%) patients. After 3 years, the number of recanalized GSV increased in 34 (7%) patients.

In the 3^rd^ group of patients (reflux only in the SFJ and GSV in competent AAGSV), after 3 months, there was a recurrence of VD due to AAGSV failure in 5 (1.5%) cases, after 6 months – in 9 (2.8%) patients. Subsequently, a year later, the failure of AAGSV caused VD recurrence in 12 (3.7%) patients, after 2 years – in 17 (5.2%) patients, and after 3 years – in 21 (6.5%) cases. It is noteworthy that VD recurrence occurred not only in the AAGSV. Conglomerates of varicose veins were observed in the GSV in the lower third of the thigh and the SSV in 34 (10.5%) patients due to vertical and horizontal reflux. To eliminate the causes of VD recurrence in this group of patients, a set of minimally invasive surgeries depending on clinical and sonographic examinations were performed. In particular, exclusion of the AAGSV from the bloodstream was performed according to the method described as well as the RFA for thermal ablation of the SSV, mini phlebectomy of dilated venous inflows. Treatment of failed perforants was carried out by foam ECHO-sclerotherapy with 1% solution of polidocanol, and obligatory preliminary tumescence and washing of the perforating vein area with physiological solution cooled to +1–3°C for its angiospasm or by perforating vein RFA with a stiletto electrode 6 Fr (2.0 mm) according to the standard techniques.

**Figure 3.**
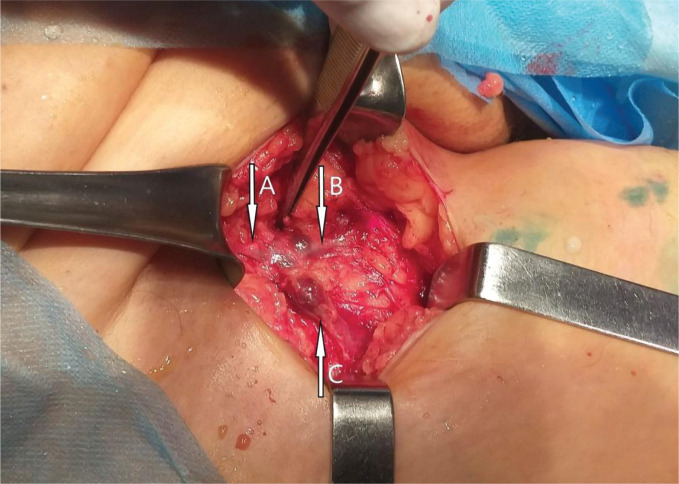
High fusion of the AAGSV with GSV directly in SFJ (A – SFJ, B – GVS, C – AAGSV).

The analysis of the causes of varicose disease recurrence showed that it was caused by: a) high fusion of the AAGSV with GSV directly in the SFJ (Figure 3), which was not revealed by preoperative ultrasound (1.7%); a long stump of the GSV after the RFA (7.8%); progression of varicose disease with the SSV and formation of new reflux associated with insufficiency of the sapheno-popliteal junction (SPJ) (4.7%); d) insufficiency of perforating veins of the tibioperoneal group (Sherman, Boyd), as well as Gunter (3.8%); e) neovascularization with dilation of small vessels in the area of the sapheno-femoral junction (0.97%).

The effectiveness of surgeries was evaluated involving 115 patients, who underwent only RFA GVS (the 1^st^ group), and 131 patients (the 2^nd^ group) who underwent both RFA GVS and AAGSV (Table 1). The assessment of clinical severity before surgery showed: the venous clinical severity score (VCSS) (C) in all patients was (6.1±0.2) points, the venous segmental disease score (VSDS) (A) – (2.09±0.12) points, the venous disability score (VDS) (D) – (1.9±0.10) points (p<0.001). The average severity of chronic venous disease (VSS) (C+A+D) before surgery was (10.2±0.2) points (p<0.001). After 6 surgeries, a significant (p<0.001) decrease in the severity of the disease was observed in the 2^nd^ group of patients (3.18±0.12), compared to 4.0±0.2 in the 1^st^ group. A year later, there was a difference in the VSS in both groups of patients. Thus, it was established that the severity of chronic venous disease was reduced in the 2^nd^ group of patients than in the 1^st^ group. In 3 years, this difference was significant: (4.8±0.2) compared to (6.4±0.3) points (p<0.001). This statistically significant difference in the VSS between the groups of patients who underwent surgery can be explained by the predominance of the number of VD recurrences in the patients who underwent only RFA of GSV and associated clinical manifestations of recurrence.

**Table 1.** Quantitative assessment of the effectiveness of surgery for varicose disease of the lower extremities.

**Parameters**	**Before surgery**	**Late postoperative period**					
		**In 6 months**		**In 1 year**		**In 3 years**	
		**RFA GSV**	**RFA GSV+AAGSV**	**RFA GSV**	**RFA GSV+AAGSV**	**RFA GSV**	**RFA GSV+AAGSV**
**VSDS (A)**	2.09±0.12	0.31±0.2*	0.28±0.1*	0.38±0.2*	0.32±0.1*	1.4±0.2*	0.9±0.2*
**VCSS (C)**	6.1±0.2	2.4±0.1*	1.9±0.2*	2.9±0.1*	2.2±0.2*	3.4±0,3*	2.6±0.2*
**VDS (D)**	1.9±0.3	1.3±0.2*	1.0±0.2*	1.4±0.1*	1.2±0.2*	1.6±0.3*	1.3±0.1*
**VSS**	10.2±0.2	4.0±0.2*	3.18±0.12*	4.68±0.1*	3.78±0.2*	6.4±0.3*	4.8±0.2*

* – p<0.001 compared to the control group.

A comparative assessment of QL after different surgery methods was carried out 3 years after their implementation (Table 2). Thus, QL in all patients who underwent surgery significantly improved than before surgery. However, after the RFA GSV+AAGSV, the patients had better QL on all scales than those who underwent only RFA GSV.

**Table 2.** Quality of life of the patients in 3 years after the RJA GSV and RJA GSV+AAGSV.

**Scales**	**Surgeries in varicose disease**		
	**Before surgery (n=55)**	**RFA GSV+AAGSV (n=110)**	**RFA GSV (n=98)**
**Physical Functioning**	79.6±0.8	89.8±0.6*	83.7±0.5*
**Role-Physical Functioning**	75.2±0.7	82.4±0.7*	79.3±0.7*
**Bodily pain**	84.7±0.3	89.4±0.8*	84.6±0.3*
**General Health**	74.3±0.6	92.05±0.7*	89.5±0.4*
**Vitality**	82.5±0.4	90.05±0.4*	88.7±0.5*
**Social Functioning**	81.7±0.6	92.08±0.1*	90.3±0.6*
**Role-Emotional**	77.6±0.8	89.8±0.7*	87.8±0.1*
**Mental Health**	75.9±0.5	90.1±0.6*	87.4±0.4*

* – p<0.05 compare to the group of patients before the surgery.

Considering the analysis of quantitative data on the effectiveness of surgeries in varicose disease and the quality of life of patients, we concluded that the surgeries performed simultaneously for the GSV and AAGSV have better functional results in the late postoperative period than the RFA only for the GSV. This is explained by an increase in the number of venous recurrences with thermal ablation for only the GSV that necessitates additional surgery for the AAGSV, dilated venous inflows, SSV, and perforating veins.

## Discussion

Our study establishes that the frequency of isolated reflux in the AAGSV is much higher than described in the literature [17–19]. It was established that its frequency was 12%. The results of our study confirm the opinion of several scientists that the AAGSV may cause recurrence of varicose veins in SFJ insufficiency [20–24]. Thus, recurrence of VD was evidenced in 20.7% of patients after surgery only in the incompetent AAGSV. Also, recurrence of the incompetent AAGSV was one of the main causes of VD recurrence in 11.7% of patients after the RFA GSV in the late postoperative period. Thus, tactical and technical approaches to diagnosing and treating VD using thermal ablation methods should be improved. In particular, attention should be paid to the individual anatomical features in every patient during SFJ and SPJ ultrasound [25–27]: the state of the terminal and preterminal valve; the presence of reflux in the SFJ; the location of additional anterior inflows of the GSV and their distance to the SFJ; insufficiency of the SPJ and perforating veins.

Regarding technical aspects of varicose disease recurrence prevention, the RFA technology allowed placing the ClosureFast catheter head directly under the GSV terminal valve, leaving the minimum length of its stump and using the GSV segmental ablation technology with automatic setting of radiofrequency energy power parameters depending on the diameter of the vein. The adjacent location of the ClosureFast catheter allowed excluding additional GSV tributaries from the bloodstream. The tributaries were located as follows: anterior GSV tributary, which flowed into the lateral wall of the GSV; the front inflow of GSV, which flowed into the front wall of the GSV; anterior tributary of the GSV, which flowed into the posterior wall of the GSV [28]. Thus, this allowed minimizing recurrence from the SFJ to the GSV tributaries and the GSV. In the mouth diameter of the GSV of more than 16±0.4 mm, 3–4 cycles of irradiation were applied, which allowed complete ablation of its lumen.

In cases of a specific anatomical situation, duplex in ultrasound examination of the AAGSV characterized by the fusion of the AASGV with the GSV from the SFJ, crossectomy combined with ligation of the GSV tributaries from RFA of the GSV trunk was used. This was also applied regarding the recurrence of VD caused by this anatomical feature.

We agree with other research that real data on recurrence in the GSV and AAGSV after radiofrequency ablation can be attained after long-term clinical and ultrasound follow-up of patients who underwent surgery [28, 29]. Thus, during the first months of follow-up, a small percentage of recurrences of VD, *i.e.* 3.3% of all patients who underwent surgery, was evidenced; and in the late postoperative period (up to 3 years of follow-up), this percentage was 11.5%. We favor "aggressive" treatment of VD recurrence in the early stages of its development to prevent its progression. Thus, if in dynamic ultrasound, in 2–3 months after the surgery, the signs of insufficiency of the AAGSV or GSV tributaries are revealed, delayed surgery should be performed: introduction of foam sclerosant in cases of the AAGSV diameter of up to 3–4 mm, or cases of the RFA diameter of more than 4–5 mm. Varicose transformed GSV tributaries are eliminated by miniphlebnetomy according to the Varadi method or foam sclerotherapy. The need for delayed surgery should be discussed with the patient before treating varicose disease.

We understand that treatment of postoperative recurrence of varicose disease is quite challenging. Therefore, in case of late recurrence of varicose disease, a set of minimally invasive surgeries should be applied according to the obtained clinical and ultrasound findings. It should be taken into account that the main factors of recurrence of VD after surgery are perforating veins, recanalized GSV, new AAGSV reflux, SSV reflux associated with SPJ insufficiency, and reflux in the perforating veins of the leg, thigh. Our results correspond with the conclusions of other authors on the factors of VD recurrence [29].

## Conclusion

Furthermore, simultaneous thermal ablation of the AAGSV and GSV combined with other minimally invasive interventions significantly reduces the percentage of postoperative recurrence of varicose disease. Therefore, there is a debatable issue of great significance that is the implementation of simultaneous RFA AAGSV, GSV/SSV aimed at "anticipating" development of recurrence of VD; or should RFA GSV/SSV be followed by dynamic sonographic monitoring of the AAGSV and collateral veins to perform delayed surgery for signs of venous insufficiency? Unfortunately, the answers to these questions have not been presented in the available literature, and they are a matter for further research.

## Acknowledgments

### Conflict of interest

The authors declare no conflict of interest.

### Ethical approval

The study was approved by the Meeting of the Bioethics Commission of I. Horbachevsky Ternopil National Medical University of the Ministry of Health of Ukraine (No. 66a/November 01/2021).

### Consent to participate

Written informed consent was obtained from the participants in the study.

### Authorship

VG contributed to conceptualization, visualization, supervision, writing, reviewing, and editing.

US contributed to the methodology, software, and guarantor of the study. OP contributed to data analysis and interpretation. BM contributed to formal analysis and investigation. 
